# Robust Protection of III–V Nanowires in Water Splitting by a Thin Compact TiO_2_ Layer

**DOI:** 10.1021/acsami.1c03903

**Published:** 2021-06-23

**Authors:** Fan Cui, Yunyan Zhang, H. Aruni Fonseka, Premrudee Promdet, Ali Imran Channa, Mingqing Wang, Xueming Xia, Sanjayan Sathasivam, Hezhuang Liu, Ivan P. Parkin, Hui Yang, Ting Li, Kwang-Leong Choy, Jiang Wu, Christopher Blackman, Ana M. Sanchez, Huiyun Liu

**Affiliations:** † Department of Electronic and Electrical Engineering, University College London, London WC1E 7JE, U.K.; ‡ Department of Physics, University of Warwick, Coventry CV4 7AL, U.K.; § Department of Chemistry, University College London, London WC1H 0AJ, U.K.; ∥ Institute of Fundamental and Frontier Sciences, University of Electronic Science and Technology of China, Chengdu 610054, P. R. China; ⊥ UCL Institute for Materials Discovery, University College London, Roberts Building, Malet Place, London WC1E 7JE, U.K.; # Department of Materials, Imperial College London, Exhibition Road, London SW7 2AZ, U.K.; ∇ Institute of Biomedical Engineering, Chinese Academy of Medical Sciences & Peking Union Medical College, Tianjin 300192, P. R. China; ○ Department of Physics, Paderborn University, Warburger Straße 100, 33098 Paderborn, Germany

**Keywords:** III−V nanowires, narrow-band-gap semiconductors, water splitting, thin TiO_2_ protection, long-term stability

## Abstract

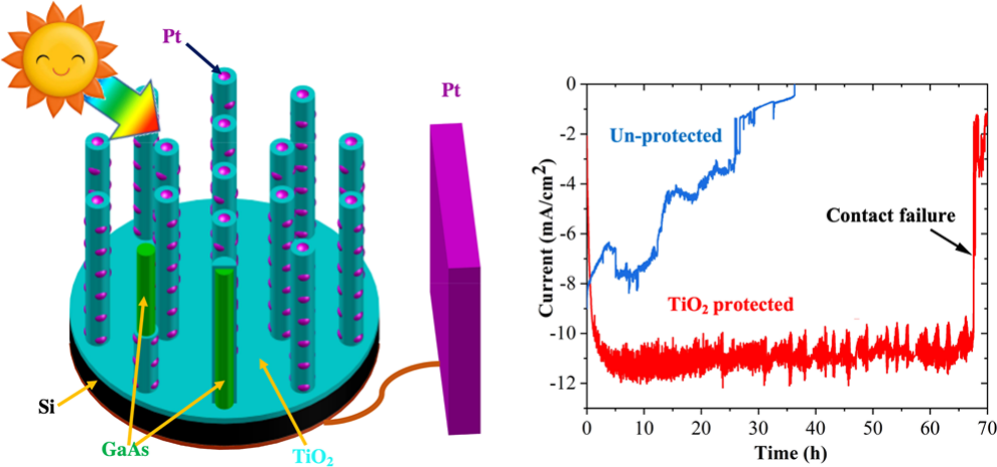

Narrow-band-gap III–V semiconductor nanowires (NWs) with a suitable band structure and strong light-trapping ability are ideal for high-efficiency low-cost solar water-splitting systems. However, due to their nanoscale dimension, they suffer more severe corrosion by the electrolyte solution than the thin-film counterparts. Thus, short-term durability is the major obstacle for using these NWs for practical water-splitting applications. Here, we demonstrated for the first time that a thin layer (∼7 nm thick) of compact TiO_2_ deposited by atomic layer deposition can provide robust protection to III–V NWs. The protected GaAs NWs maintain 91.4% of its photoluminescence intensity after 14 months of storage in ambient atmosphere, which suggests the TiO_2_ layer is pinhole-free. Working as a photocathode for water splitting, they exhibited a 45% larger photocurrent density compared with unprotected counterparts and a high Faraday efficiency of 91% and can also maintain a record-long highly stable performance among narrow-band-gap III–V NW photoelectrodes; after 67 h photoelectrochemical stability test reaction in a strong acid electrolyte solution (pH = 1), they show no apparent indication of corrosion, which is in stark contrast to the unprotected NWs that fully failed after 35 h. These findings provide an effective way to enhance both stability and performance of III–V NW-based photoelectrodes, which are highly important for practical applications in solar-energy-based water-splitting systems.

## Introduction

Solar energy is abundant, clean, and renewable, which makes it one of the most promising renewable energy sources that can solve the worldwide energy crisis and the serious environmental problems caused by the combustion of fossil fuels.^1^ Solar-driven water splitting can harvest solar energy and directly convert it into chemical energy, such as hydrogen from water reduction.^2^ Dihydrogen has high energy density, which is beneficial for energy storage and transportation, and is particularly attractive as an energy carrier.^3−5^ The combustion of hydrogen to water does not cause any environmental pollution. Hydrogen generation by photoelectrochemical (PEC) water splitting has thus gained great attention.^4,6−9^

PEC water splitting requires thermodynamic potentials of ∼1.23 and ∼0.95 eV for full and Z-scheme half water splitting, respectively.^10−12^ A potential much larger than these values will lead to low energy conversion efficiency due to energy loss.^10^ For example, ZnO with a large band gap of 3.37 eV can only covert a very small portion of high-energy photons in the solar spectrum. Thus, the high-efficiency semiconductor photoelectrode needs to have a narrow band gap as close as possible to these ranges to convert a larger portion of energy from the solar spectrum.^13−16^ The band gap in many III–V semiconducting materials lies in this region. Their direct band gap allows high-efficiency photon absorption, making them suitable for high-efficiency solar water splitting (∼20%).^17^ However, III–V materials are relatively rare and expensive for extensive use. Innovations are needed to harvest solar energy with greater economic viability. The ideal solution is to build the high-efficiency III–V cells onto the low-cost mature Si platform. However, after 20 years of research, the lattice and thermal expansion coefficient mismatches between III–V epilayers and Si substrates still hinder the effective implementation of this idea.^18^

III–V nanowire (NW) structures have demonstrated many novel mechanical, optical, and electronic properties that are not present in the thin-film counterparts.^19,20^ High-quality NWs can be grown on inexpensive substrates such as Si, graphene, carbon nanotubes, fiber-textured silicon thin films, amorphous Si, glass, and indium tin oxide, to significantly reduce the overall device cost.^21^ They can also behave as optical antennas^22^ to concentrate light as they can greatly enhance the light absorption cross section (up to 12 times) compared to their physical size.^23^ NW arrays can also increase light scattering due to their subwavelength dimensions,^24^ resulting in the internal light path lengths up to 73 times longer compared with that of their thin-film counterparts.^25^ Therefore, NW arrays have advanced light-trapping ability, allowing to use a small amount of expensive III–V material and achieve as efficient optical absorption as thick bulk counterparts.^26^ Moreover, the large surface-to-volume ratio of the NWs provides a lower barrier for the chemical reaction.^4^ Thus, III–V NW-based photoelectrodes for water splitting have attracted great attention.^27,28^

Severe photoinduced corrosion of III–V materials in the electrolyte solution is a common problem, and III–V NWs with a nanoscale size are even more vulnerable.^29−31^ Due to the unique one-dimensional structure, they are much more difficult to protect. The lifetime of the unprotected NW water-splitting cells is commonly less than 1 day that is far too short to have practical applications.^31^ Titanium dioxide (TiO_2_) is one of the most common protection layer materials that is stable over a wide range of pH and potentials.^18,32^ It also forms a type-II heterojunction with most III–V materials and serves as an effective hole blocking layer while allowing the transport of electrons to the surface for water splitting.^10,33^ This allows it to reduce the carrier loss by nonradiative surface recombination, and effectively improve the quantum efficiency.^32^ Thus, it has been widely used in III–V thin-film devices, and has demonstrated quite robust protection against electrolyte corrosion.^34^ When grown by atomic layer deposition (ALD), it can conformably cover the three-dimensional sample surface with a uniform and precise thickness, therefore, giving good protection to III–V NWs during the water-splitting process. ALD-deposited TiO_2_ thin films with controlled layer growth on an atomic level enable the generation of highly conformal layers, which has been widely used in a wide range of areas for various purposes, such as enhancing cycling stability and Coulombic efficiency in batteries,^35,36^ capping layer to prevent photocorrosion,^37,38^ and improving mechanical and chemical properties of 1D nanomaterials.^39,40^ In our previous work, we have grown III–V NWs with excellent optoelectronic properties. When applied for PEC water splitting, III–V NWs exhibited a series of PEC instability problems in aqueous alkaline and acidic electrolyte solutions. TiO_2_ is an n-type semiconductor with electrochemical stability in corrosive media; therefore, it is a good candidate as a protective layer for III–V nanowire-based photoelectrodes. The presence of pinholes in the protection layers is detrimental for the devices. It was shown that a thick TiO_2_ protection layer of at least 40 nm is required to achieve pinhole-free films.^32,33^ From the literature, the thickness for ALD-deposited TiO_2_ protective layers in thin-film PEC applications is usually very thick (40–100 nm) to reach long-term stability (in the range of tens of hours).^32^ NWs have a one-dimensional column structure with a high aspect ratio, which makes it much more challenging than thin films in the realization of a pinhole-free TiO_2_ protection layer. It was demonstrated that a 50 nm thick TiO_2_ passivation layer on NWs for PEC can only maintain ∼80% of the performance over only 20 h.^41^ However, increasing the thickness of the TiO_2_ layer can bring many side effects and eventually outweigh the benefits of passivation. At a wavelength range from 500 to 900 nm, the transmission of the TiO_2_ layer reduces from >90 to ∼60%, and the reflection increases from <10 to >20% when the thickness increases from 1.4 to 136 nm.^32^ The absorption of a 70.92 nm TiO_2_ layer is ∼10% at the same wavelength range.^32^ Moreover, the thick TiO_2_ layer can increase the charge transfer resistance and the electron tunneling would become unlikely if the thickness exceeds the critical thickness (e.g., 10 nm).^32,42^ It has been reported that the increase in TiO__2__ thickness can increase the overpotential with a linear rate of ∼21 mV/nm, which results in an additional voltage loss.^43−45^ Thus, it is critical for the TiO_2_ protection layer to be pinhole-free at a thin thickness. Thus, as far as we are aware, there is still a lack of reports on the achievement of a long-term stability using only a thin TiO_2_ protective layer in water splitting, especially for NW-based devices. Although a few reports on thin-film devices exhibited hours of stability for very thin TiO_2_ layers (<10 nm), they are probably supported by the interlayer of SiO_2_ or by a thicker catalyst overlayer (e.g., Ni of 7–100 nm thickness), which would add further cost and/or cause additional adverse effects.^32^ Therefore, it is important to develop a robust protection technique using only a thin (sub 10 nm) layer of TiO_2_.^46,47^^46,47^

In this study, the protective behavior of a very thin TiO_2_ layer on III–V NWs was studied in depth. With TiO_2_ protection, the GaAs NW photocathode demonstrated greatly improved performance and a record-long durability in the photoelectrochemical reaction among narrow-band-gap III–V NWs. This solves a major challenge for using narrow-band-gap III–V NWs in water splitting for environmentally clean and renewable energy generation.

## Results and Discussion

### Structural Information

**Figure 1:**
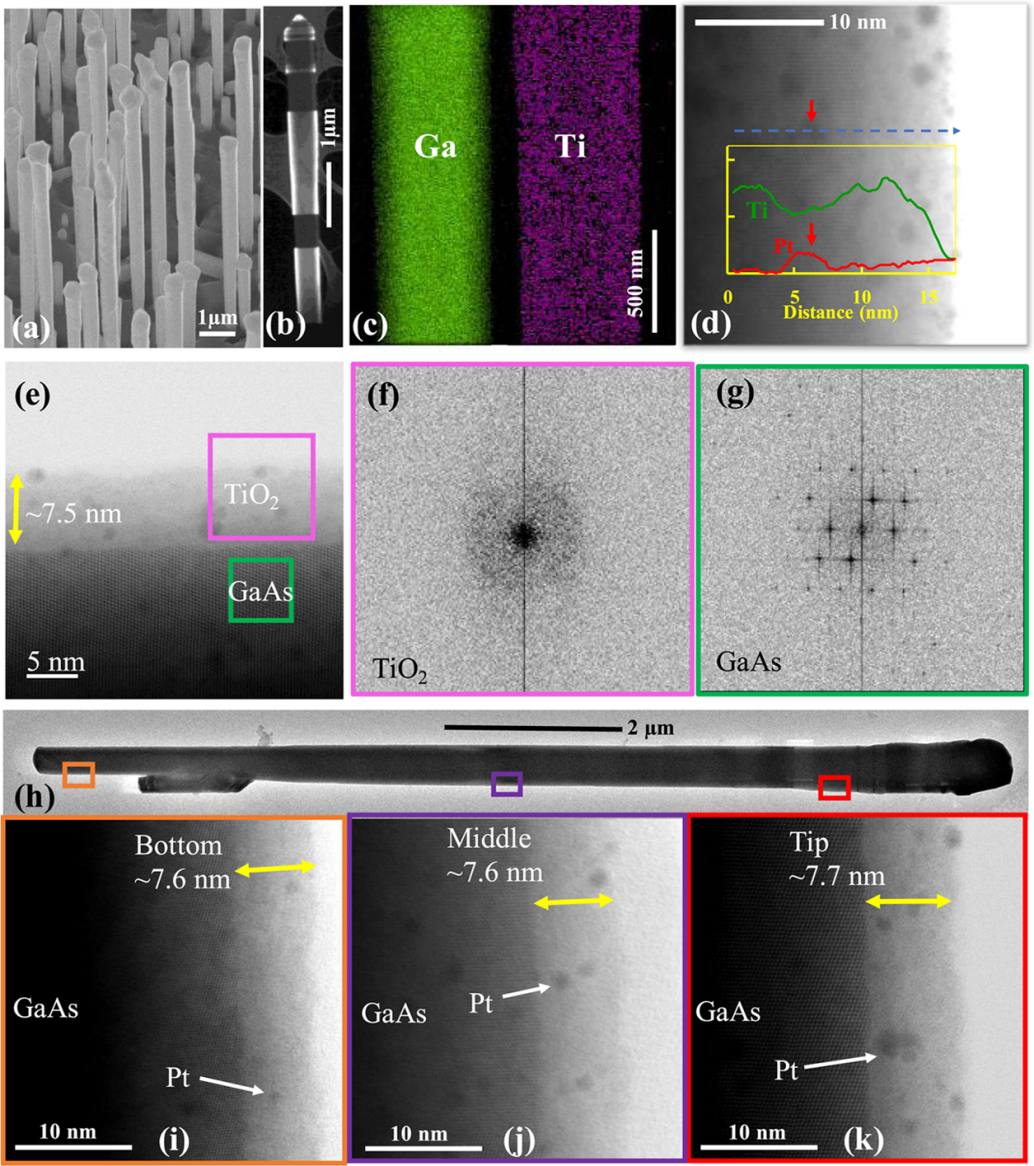
Structural information of GaAs NWs covered by TiO_2_ and Pt. (a) Low-magnification SEM images showing the NWs. (b) Low-magnification TEM image of a NW. (c) EDX mapping of Ga and Ti in a NW segment. (d) EDX composition line scan near the NW surface. (e) Higher-magnification TEM image showing the NW/TiO_2_ interface. (f) and (g) are the fast fourier transforms of areas shown in (e). (h) Low-magnification TEM image showing an entire NW. Higher-magnification TEM images showing the NW/TiO_2_ interfaces at the (i) bottom, (j) middle, and (k) tip of the NW marked in (h).

GaAs NWs were grown by molecular beam epitaxy (MBE) via self-catalyzed mode on p-type Si substrates.^48^ The majority of NWs stand vertically on the substrate as can be seen in the scanning electron microscopy (SEM) image in Figure 1a. They are ∼10 μm in length with a diameter of ∼300 nm at the bottom, which gradually increases to ∼550 nm at the tip. The lower two-thirds of the NW is the zinc blende crystal structure with occasional single twins, as can be seen in the transmission electron microscopy (TEM) image in Figure 1b. The NW tip is enlarged with an irregular shape caused by unoptimized consumption of the Ga catalytic droplet after the core-NW growth. Defect-free core–shell NWs with high crystalline quality and regular morphology can be achieved by optimized the growth parameters.^49^

NWs were then coated by a uniform layer of amorphous TiO_2_ deposited by ALD, confirmed by the energy dispersive X-ray spectroscopy (EDX) composition mapping of Ga and Ti shown in Figure 1c and the composition line scan near the surface shown in Figure 1d, as well as the X-ray photoelectron spectroscopy (XPS) spectra in Supporting Information Figure S1a. TiO_2_ is amorphous which can be seen in Figure 1e–g and in more detail in Supporting Information Figure S2, as it is in contrast to GaAs with a clear crystal lattice. The thickness of the TiO_2_ protection layer is ∼7 nm with a high uniformity from the bottom to the tip of the NWs, as shown in Figure 1e–h. Uniform deposition of conformal films with highly controllable thickness on complex three-dimensional surfaces is a key benefit of ALD growth. The choice of TiO_2_ thickness is for achieving high transmission (>90%), low reflectance (<10%), and good carrier transportation ability.^32,42^ The Pt cocatalyst was deposited on the surface of TiO_2_ by aerosol-assisted chemical vapor deposition (AACVD). Pt formed small particles (<3 nm) which are confirmed by EDX line scans in Figure 1d. The Pt nanoparticles decorate the surface of the NWs as shown in Figure 1h–k, but the density and size gradually reduce from the tip to the bottom, possibly due to the shadowing effect caused by the neighboring NWs. Pt growth by ALD can have much better coverage and probably also Pt performance. However, we study the protection effect of a thin TiO_2_ layer. A lower percentage of the surface being covered by Pt would be better for the study of this topic. Further study can be continued with ALD-grown Pt.^50^

### Compact TiO_2_ and Its Influence on Optical Properties

**Figure 2:**
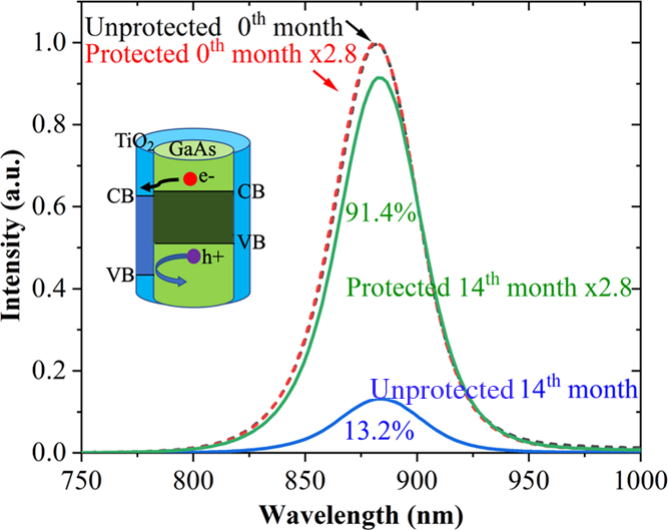
PL spectra from GaAs NWs with and without a TiO_2_ protection layer. The TiO_2_ protection layer was deposited on the same day of the NW growth. The spectra were taken on the 1st day of NW growth and after 14 months storage in atmosphere.

The influence of TiO_2_ on the optical properties of GaAs NWs was analyzed by photoluminescence (PL) spectroscopy. Figure 2 shows the PL spectrum of GaAs NWs with and without the TiO_2_ layer. With the addition of TiO_2_ on the same day after the NW growth, PL emissions from GaAs NWs are quenched by a factor of 2.8, which is typical for type-II heterojunctions that can efficiently separate charge carriers and thereby reduce radiative recombination.^10^ As illustrated in the inset of Figure 2, the conduction band (CB) of GaAs is slightly higher than that of TiO_2_; therefore, electron migration from the NWs to the surface is promoted, allowing efficient water splitting. The valence band of TiO_2_ is much lower than that of GaAs, thus forming a favorable barrier to keep holes away from the surface.

When exposed to air, the NW surface can be oxidized to form a native oxide layer (1–2 nm), which acts as a high-density nonradiative recombination center, resulting in charge trapping. This can consume a large portion of the photon-generated carriers and lead to low light emission. After storing in atmosphere for 14 months, the NWs without TiO_2_ protection experienced severe decay and the emission intensity reduced to 13.2%. In contrast, the NWs with a ∼7 nm TiO_2_ protection layer can maintain 91.4% of the emission intensity after storing in the same environment and period. This suggests that the TiO_2_ layer has good compactness that can prevent the permeation of oxygen and water, and thus can provide a long-term protection to the surface of the NWs.

### Photoelectrochemical Performance

The PEC performance of GaAs NW photoelectrodes with and without TiO_2_ was determined using them as a photocathode for water splitting and H_2_ generation. A three-electrode system, working electrode, Ag/AgCl as reference electrode, and Pt as counter electrode, was used. H_2_SO_4_ (0.5 M, pH = 1) solution was used as the electrolyte, with the PEC reaction being carried out under 1 sun illumination from AM 1.5 G solar simulator.

**Figure 3:**
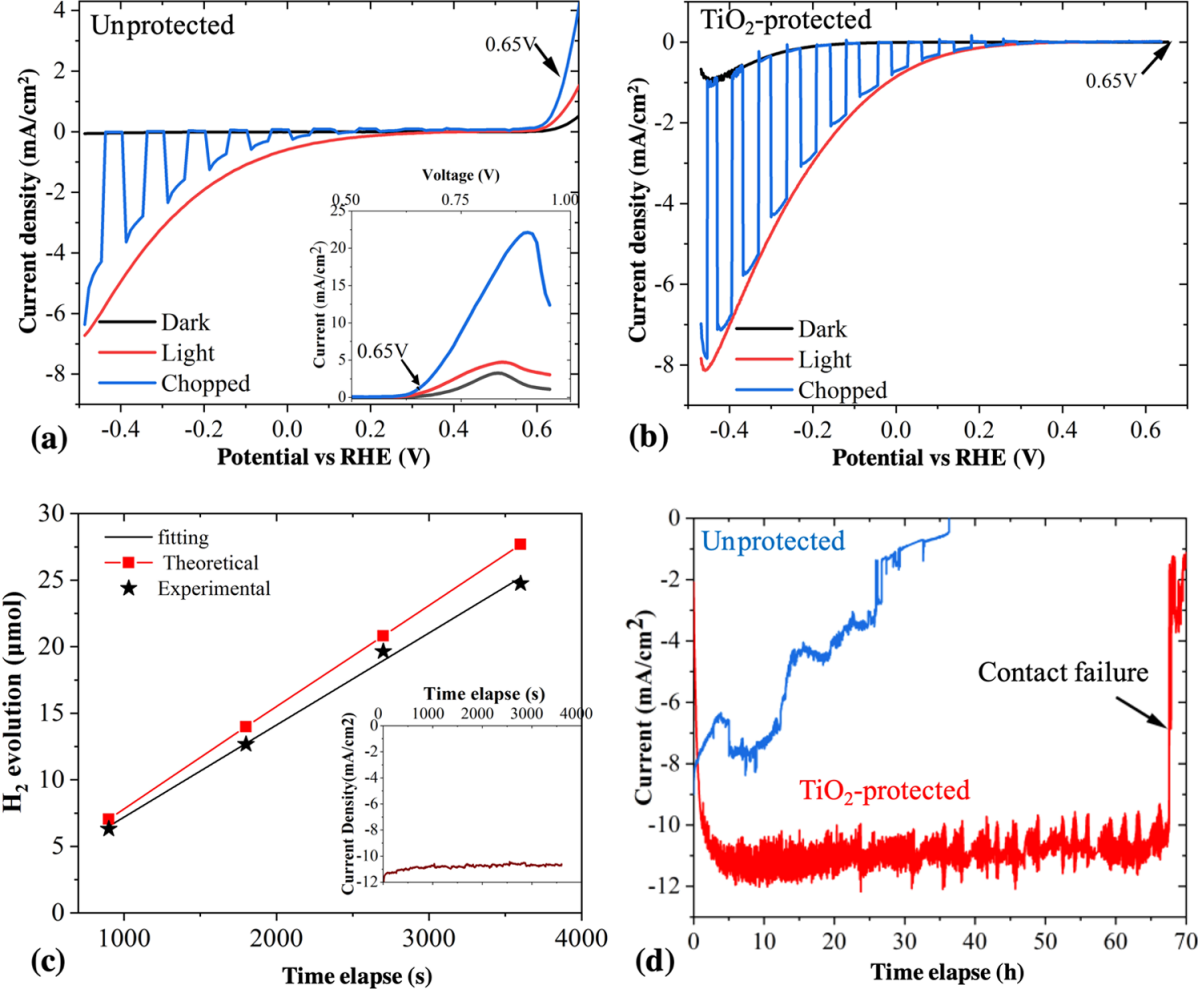
Photoelectrochemical properties of GaAs photocathodes. (a) *J*–*V* curves of unprotected photocathodes and the inset figure shows the enlarged *J*–*V* curve above 0.5 V vs RHE. (b) TiO_2_-protected photocathodes under 1 sun illumination (AM 1.5 G 100 mW/cm^2^) in 0.5 M H_2_SO_4_ electrolyte (pH = 1). (c) Experimental and theoretical H_2_ evolution of TiO_2_-protected photocathodes under continuous illumination (AM 1.5 G 100 mW/cm^2^). (d) Stability of the protected and unprotected photocathodes measured with a bias of −0.6 V and under an AM 1.5 sun illumination.

As shown in Figure 3a, the unprotected GaAs NW photocathodes have a photocurrent onset potential of ∼0.233 V reversible hydrogen electrode (RHE) and a photocurrent density of 0.598 mA/cm^2^ at 0 V vs RHE. As shown in Figure 3b, the protected GaAs NW photocathodes have a slightly larger photocurrent onset potential of ∼0.243 V RHE, and a larger photocurrent density of 0.87 mA/cm^2^ at 0 V vs RHE—a 45% increase. The increased photocurrent density is due to TiO_2_ surface passivation and the type-II band alignment between TiO_2_ and GaAs NWs that are beneficial for efficient carrier separation, as discussed above.

A gas chromatograph (GC) system was used to measure the actual H_2_ generation rate of the protected NW photocathode when under continuous irradiation with a bias voltage of −0.6 V. As shown in Figure 3c, the generated H_2_ volume increased linearly. The theoretical generation value of hydrogen is obtained from the *I* × *t* curve by the equation^51^
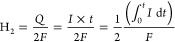
1where *F* is the Faraday constant (96 484.34 C/mol), *Q* is the amount of charge passed in time *t*, and *I* is the photocurrent. When the current is not constant, the amount of charge passing through the circuit can be estimated by integrating the current over time. The Faraday efficiency can be calculated by the equation
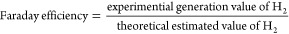
2

As can be deduced from Figure 3c, the H_2_ generation rate is about 22 μmol/h and is highly stable for the 1 h period measured, which can be further supported by the stable photocurrent in the inset. The photocathode also exhibited a high and stable Faraday efficiency of 91%, which shows that the surface condition is highly favorable for carriers to participate in the water-splitting reaction.

### Photoelectrochemical Stability

The corrosion current of the unprotected NW photocathode is ∼2 mA/cm^2^ at 0.65 V vs RHE and increases rapidly with a forward bias as can be seen in Figure 3a and its inset; however, the TiO_2_-protected photocathodes do not show photocorrosion current at 0.65 V vs RHE, which suggests better stability in the PEC reaction. To further confirm this, a stability test of the hydrogen evolution reaction of the photocathodes was carried out. Hydrogen is uniformly generated on the surface of the test samples, and the hydrogen generation rate is fast and stable when the NWs are in a good state (Supporting Information S3). As shown in Figure 3d, the current density of the unprotected NW photocathode started at about 8.5 mA/cm^2^ under −0.6 V vs RHE and then dropped rapidly to ∼0 mA/cm^2^ after ∼36 h, while the initial current density of the protected photocathode was ∼11 mA/cm^2^ and highly stable over a long duration of 67 h that is already much longer than other narrow-band-gap III–V NW photoelectrodes as far as we know. Beyond this time, no data is available due to the failure of the back contact (Supporting Information S4). The epoxy was immersed in the corrosive electrolyte for a long time, resulting in leakage at the connection with the electrode, which causes the electrolyte solution to penetrate into the back contact and then the electrode fails.

**Figure 4:**
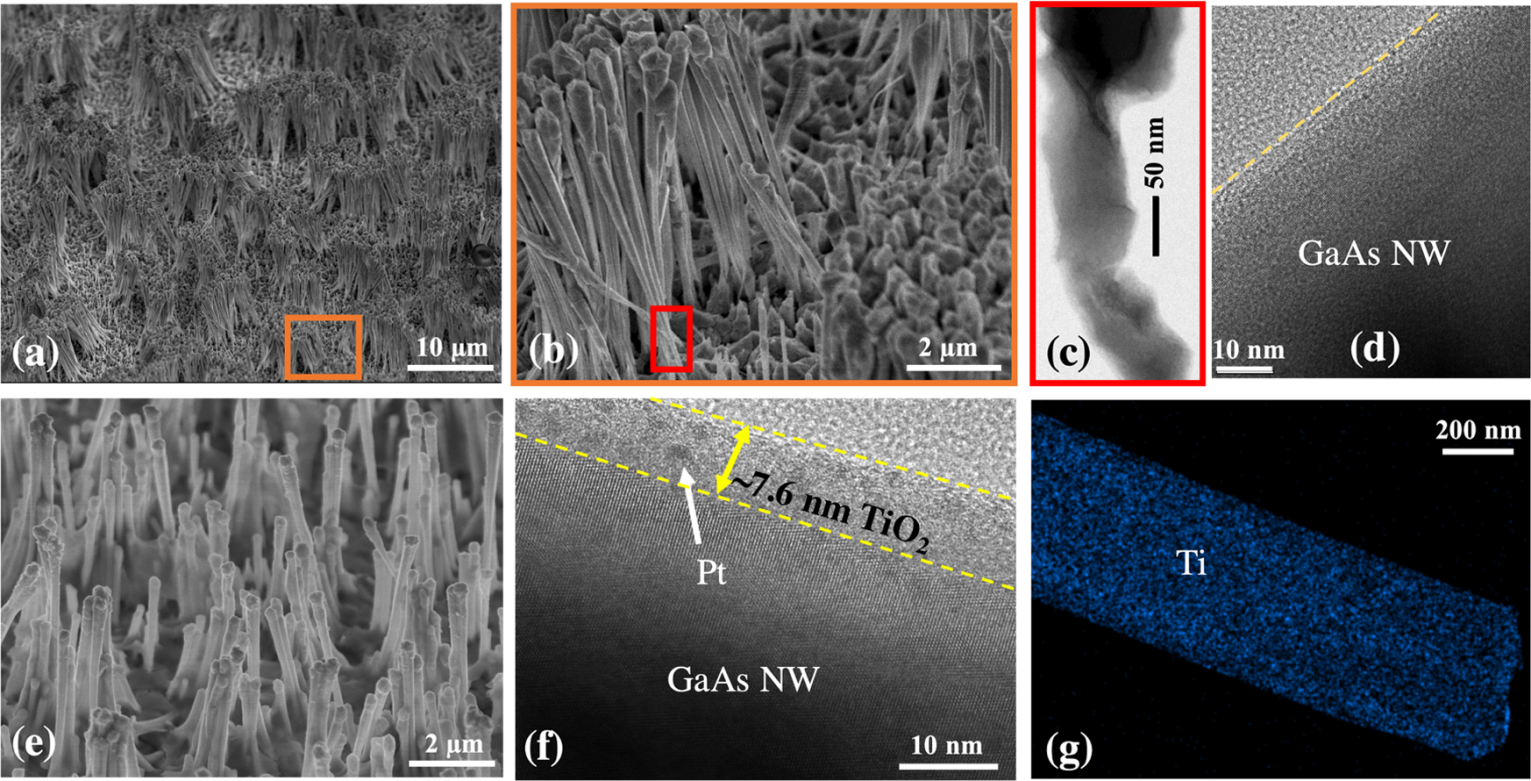
Morphology and structure of GaAs NW photocathodes after the stability test. (a–d) are from unprotected photocathode. (a) SEM image gives an overall view of the sample surface. (b) Higher-magnification SEM image of the orange square shown in (a). (c) TEM image showing the bottom of a NW with severe corrosion. (d) TEM image showing the Pt-free surface of the NWs. (e–h) are from protected photocathodes. (e) SEM image of the sample surface. (f) TEM image of the NW surface showing GaAs/TiO_2_ interface and Pt particles. (g) Ti element mapping of the NW bottom.

After the stability test, the NWs from the unprotected photocathode were found to have detached from the substrate and bundled together, forming large NW groups and producing areas free of NWs, as can be seen in Figure 4a,b. The entire NW has been etched for the unpassivated NWs. The etching is more severe when close to the bottom part of the NW (see Figure 4b,c). This phenomenon should be due to the higher current density toward the bottom of nanowires, which has led to especially severe etching at the very bottom of the NWs. As can be seen in more detail in Figure 4c, the bottom of the NWs is highly rough and significantly thinned down to ∼50 nm due to corrosion. NWs with a high aspect ratio (i.e., ratio between length and diameter, length/diameter) tend to bend and bundle together after removal from the liquid due to the capillary force.^52^ When the NW bottom is very thin, they can break off the substrate when bending. In addition, the other parts of the NWs are also corroded during the PEC reaction, and the Pt particles seem to have been washed away as they were undermined during the severe corrosion on the uncoated NW surfaces, which can be seen in Figure 4d. For the protected photocathode, NWs are also bent and bundled, forming smaller groups of NWs as can be seen in Figure 4e. However, none of the NWs are observed to be broken off from the substrate due to solution etching. The TiO_2_ protection layer is still on the surface of the NWs with a thickness of ∼7 nm (Figure 4f), which can be further confirmed by the uniform distribution of Ti from the EDS mapping shown in Figure 4g that was taken at the bottom of the NWs. There is no noticeable change in chemical format of TiO_2_ films during PEC water splitting (Supporting Information S1). In addition, the Pt particles are also still present on the surface as can be seen in Figure 4f. Thus, no apparent corrosion from protected NWs was observed.

## Conclusions

In this study, the stability of unprotected and protected narrow-band-gap III–V NWs in the PEC reaction was investigated in detail using GaAs NWs. The unprotected NWs suffered from severe corrosion, leading to full failure in the PEC reaction within 35 h. A TiO_2_ layer of ∼7 nm was used to protect the NWs and to minimize the detrimental corrosion effects. The ALD growth can conformably cover the entire NW surface with a highly uniform TiO_2_ layer. After storing in atmosphere for 14 months, the protected NWs can still retain 91.4% of its emission intensity, outperforming the unprotected ones that can only maintain 13%. This suggests that the compact TiO_2_ film can prevent oxygen and water permeation to the surface of the GaAs NWs. The protected GaAs NWs were used as the cathode for PEC water splitting and the photocurrent density improved by 45% to 0.87 mA/cm^2^ at 0 V vs RHE due to the surface passivation effect from the TiO_2_ layer and the favorable band alignment between GaAs and TiO_2_, which improved the charge transfer and reduced the charge recombination at the photocathode. After 67 h of the PEC reaction under continuous simulated solar light illumination, there was no obvious decay of the electrode and the protection layer is still in a very good state. The protected photocathode also shows good faradic efficiency of ∼91%, which suggests that the surface condition is highly favorable for carriers to participate in the water-splitting reaction. These results show that a thin layer of compact TiO_2_ can provide superior protection to GaAs NWs and address, for the first time, the short stability issue of narrow-band-gap III–V NWs, allowing them to be practically used in the PEC reaction for a significantly longer time.

## Methods

### NW Growth

The self-catalyzed GaAs NWs were grown directly on p-type Si(111) substrates by solid-source III–V molecular beam epitaxy.^53^ The Ga beam equivalent pressure, V/III flux ratio, and substrate temperature were 8.41 × 10^–8^ Torr, ∼50, and ∼630 °C, respectively. To grow the shell, the Ga droplets were consumed by closing the Ga flux and keeping the group-V fluxes open after the growth of the core. The shells were grown with a Ga beam equivalent pressure, V/III flux ratio, substrate temperature, and growth duration of 8.41 × 10^–8^ Torr, 86, ∼500 °C, and 90 min, respectively. The substrate temperature was measured by a pyrometer. The doping concentrations of GaAs p-core, p-shell, and n-shell were 1.6 × 10^18^ (Be), 1.6 × 10^18^ (Be), and 1 × 10^18^–1 × 10^19^ (Si) cm^–3^, respectively. The thickness ratio of the GaAs p-core, p-shell, and n-shell is 2:3:3. On the p–i–n junction, an ∼30 nm Al_0.5_Ga_0.5_As surface passivation layer and an ∼10 nm GaAs protection layer were grown with a Si doping concentration of 1 × 10^19^ cm^–3^.

### Scanning Electron Microscopy (SEM)

The NW morphology was characterized with a Zeiss XB 1540 FIB/SEM system.

### Transmission Electron Microscopy (TEM)

Simple scraping of the NWs onto a holey carbon support was used to prepare TEM specimens. The TEM measurements were performed with a doubly corrected ARM200F microscope, operating at 200 kV.

### TiO_2_ Deposition

A TiO_2_ thin layer was deposited using a Savannah atomic layer deposition S100 system from Ultratech.^54^ Tetrakis(dimethylamino)titanium (TDMAT) and DI water were used as precursors for Al and O, respectively. N_2_ (CP Grade) from BOC with purity 99.9992% was used as the carrier and purging gas. Deposition was processed at 100 °C at a pressure of 1 × 10^–2^ mbar, and one ALD growth cycle was defined by the following sequence for 15 cycles (0.48 nm per cycle): TDMAT pulse (0.1 s)–N_2_ purge (5 s)–H_2_O pulse (0.015 s)–N_2_ purge (5 s).

### Pt Deposition

Pt was applied as a cocatalyst deposited on the surface of GaAs nanowires coated with TiO_2_ by the AACVD method to accelerate the hydrogen evolution reaction (HER) rate.^55−57^ Aerosols of Pt precursor dissolved in methanol were generated by an ultrasonic humidifier and then the aerosols were transported to the reactor using nitrogen carrier gas operated by a mass flow controller.^58,59^ Deposition was carried out at 350 °C with deposition time of 30 min.

### Photoelectrochemical (PEC) Measurements

The PEC properties of the GaAs nanowire photoelectrodes were measured by a three-electrode system composed of a Ag/AgCl reference electrode, a Pt counter electrode, and GaAs NWs as the photoactive working electrode. The constant potential of the working electrode was controlled by a potentiostat. The electrolyte, 0.5 M H_2_SO_4_ (pH = 1), was used as the PEC reaction solution, and the PEC reaction was carried out under 1 AM 1.5 G sun illumination. The stability test of the photocathode was carried out under an AM 1.5 sun illumination. The stability reaction was carried out in a 0.5 mol/L sulfuric acid solution (pH = 1), with a constant potential relative to an RHE equal to 0. A gas chromatograph (GC; Shimadzu GC-2014) was used to measure the hydrogen generation rate. According to the Nernst equation, the measured potential (vs Ag/AgCl) can be converted into a reversible hydrogen electrode (NHE at pH = 1): *E*_RHE_ = *E*_Ag/AgCl_ + *E*_0 Ag/AgCl_ + 0.059 × pH. Generally, at room temperature, *E*_0 Ag/AgCl_ = 0.197 V.^60^ To control photocorrosion, both the PEC test and the stability test voltage were selected as the safe voltage range, −0.733–0.4 V. The photocurrent density (*J*) and electric potential (*V*) for a GaAs nanowire Si/GaAs-TiO_2_-Pt photocathode were measured by linear scanning voltammetry. The measurement was carried out in the dark, in a chopper, and in continuous light under 1 sun illumination (AM 1.5 G 100 mW/cm^2^).^61^

## Supporting Information Available

Hydrogen generation during the stability test (MP4)
Chemical properties of TiO_2_; amorphous structure of TiO_2_; H_2_ generation during PEC reaction; and device state before and after the PEC reaction (PDF)

## References

[ref1] Headley S. A New Day Dawning. Youth Stud. Aust. 2013, 32, 1–2. 10.1038/443019a.

[ref2] Bensaid S.; Centi G.; Garrone E.; Perathoner S.; Saracco G. Towards Artificial Leaves for Solar Hydrogen and Fuels from Carbon Dioxide. ChemSusChem 2012, 5, 500–521. 10.1002/cssc.201100661. 22431486

[ref3] Fujishima A.; Honda K. Electrochemical Photolysis of Water at a Semiconductor Electrode. Nature 1972, 238, 37–38. 10.1038/238037a0. 12635268

[ref4] Walter M. G.; Warren E. L.; McKone J. R.; Boettcher S. W.; Mi Q.; Santori E. A.; Lewis N. S. Solar Water Splitting Cells. Chem. Rev. 2010, 110, 6446–6473. 10.1021/cr1002326. 21062097

[ref5] Jeon K. J.; Moon H. R.; Ruminski A. M.; Jiang B.; Kisielowski C.; Bardhan R.; Urban J. J. Air-Stable Magnesium Nanocomposites Provide Rapid and High-Capacity Hydrogen Storage without Using Heavy-Metal Catalysts. Nat. Mater. 2011, 10, 286–290. 10.1038/nmat2978. 21399630

[ref6] Chen J.; Yang D.; Song D.; Jiang J.; Ma A.; Hu M. Z.; Ni C. Recent Progress in Enhancing Solar-to-Hydrogen Efficiency. J. Power Sources 2015, 280, 649–666. 10.1016/j.jpowsour.2015.01.073.

[ref7] Landman A.; Dotan H.; Shter G. E.; Wullenkord M.; Houaijia A.; Maljusch A.; Grader G. S.; Rothschild A. Photoelectrochemical Water Splitting in Separate Oxygen and Hydrogen Cells. Nat. Mater. 2017, 16, 646–651. 10.1038/nmat4876. 28272504

[ref8] Bae D.; Seger B.; Vesborg P. C. K.; Hansen O.; Chorkendorff I. Strategies for Stable Water Splitting: Via Protected Photoelectrodes. Chem. Soc. Rev. 2017, 46, 1933–1954. 10.1039/c6cs00918b. 28246670

[ref9] Khaselev O.; Turner J. A. A Monolithic Photovoltaic-Photoelectrochemical Device for Hydrogen Production via Water Splitting. Science 1998, 280, 425–427. 10.1126/science.280.5362.425. 9545218

[ref10] Butson J. D.; Narangari P. R.; Lysevych M.; Wong-Leung J.; Wan Y.; Karuturi S. K.; Tan H. H.; Jagadish C. InGaAsP as a Promising Narrow Band Gap Semiconductor for Photoelectrochemical Water Splitting. ACS Appl. Mater. Interfaces 2019, 11, 25236–25242. 10.1021/acsami.9b06656. 31265227

[ref11] Ros C.; Andreu T.; Morante J. R. Photoelectrochemical Water Splitting: A Road from Stable Metal Oxides to Protected Thin Film Solar Cells. J. Mater. Chem. A 2020, 8, 10625–10669. 10.1039/d0ta02755c.

[ref12] Zheng J.; Zhou H.; Zou Y.; Wang R.; Lyu Y.; Jiang S. P.; Wang S. Efficiency and Stability of Narrow-Gap Semiconductor-Based Photoelectrodes. Energy Environ. Sci. 2019, 12, 2345–2374. 10.1039/c9ee00524b.

[ref13] Abdi F. F.; Han L.; Smets A. H. M.; Zeman M.; Dam B.; Van De Krol R. Efficient Solar Water Splitting by Enhanced Charge Separation in a Bismuth Vanadate-Silicon Tandem Photoelectrode. Nat. Commun. 2013, 4, 2195 10.1038/ncomms3195. 23893238

[ref14] Fountaine K. T.; Lewerenz H. J.; Atwater H. A. Efficiency Limits for Photoelectrochemical Water-Splitting. Nat. Commun. 2016, 7, 13706 10.1038/ncomms13706. 27910847 PMC5146289

[ref15] Britto R. J.; Young J. L.; Yang Y.; Steiner M. A.; Lafehr D. T.; Friedman D. J.; Beard M.; Deutsch T. G.; Jaramillo T. F. Interfacial Engineering of Gallium Indium Phosphide Photoelectrodes for Hydrogen Evolution with Precious Metal and Non-Precious Metal Based Catalysts. J. Mater. Chem. A 2019, 7, 16821–16832. 10.1039/c9ta05247j.

[ref16] Jiang C.; Moniz S. J. A.; Wang A.; Zhang T.; Tang J. Photoelectrochemical Devices for Solar Water Splitting-Materials and Challenges. Chem. Soc. Rev. 2017, 46, 4645–4660. 10.1039/c6cs00306k. 28644493

[ref17] Ben-Naim M.; Britto R. J.; Aldridge C. W.; Mow R.; Steiner M. A.; Nielander A. C.; King L. A.; Friedman D. J.; Deutsch T. G.; Young J. L.; Jaramillo T. F. Addressing the Stability Gap in Photoelectrochemistry: Molybdenum Disulfide Protective Catalysts for Tandem III–V Unassisted Solar Water Splitting. ACS Energy Lett. 2020, 5, 2631–2640. 10.1021/acsenergylett.0c01132.

[ref18] Tournet J.; Lee Y.; Karuturi S. K.; Tan H. H.; Jagadish C. III–V Semiconductor Materials for Solar Hydrogen Production: Status and Prospects. ACS Energy Lett. 2020, 5, 611–622. 10.1021/acsenergylett.9b02582.

[ref19] Lieber C. M.; Wang Z. L. Functional Nanowires. MRS Bull. 2007, 32, 99–108. 10.1557/mrs2007.41.

[ref20] Yang P.; Yan R.; Fardy M. Semiconductor Nanowire: Whats Next?. Nano Lett. 2010, 10, 1529–1536. 10.1021/nl100665r. 20394412

[ref21] Lohn A. J.; Li X.; Kobayashi N. P. Epitaxial Growth of Ensembles of Indium Phosphide Nanowires on Various Non-Single Crystal Substrates Using an Amorphous Template Layer. J. Cryst. Growth 2011, 315, 157–159. 10.1016/j.jcrysgro.2010.08.050.

[ref22] Van Dam D.; Abujetas D. R.; Paniagua-Domínguez R.; Sánchez-Gil J. A.; Bakkers E. P. A. M.; Haverkort J. E. M.; Gómez Rivas J. Directional and Polarized Emission from Nanowire Arrays. Nano Lett. 2015, 15, 4557–4563. 10.1021/acs.nanolett.5b01135. 26043200

[ref23] Krogstrup P.; Jørgensen H. I.; Heiss M.; Demichel O.; Holm J. V.; Aagesen M.; Nygard J.; Fontcuberta I Morral A. Single-Nanowire Solar Cells beyond the Shockley-Queisser Limit. Nat. Photonics 2013, 7, 306–310. 10.1038/nphoton.2013.32.

[ref24] Muskens O. L.; Rivas J. G.; Algra R. E.; Bakkers E. P. A. M.; Lagendijk A. Design of Light Scattering in Nanowire Materials for Photovoltaic Applications. Nano Lett. 2008, 8, 2638–2642. 10.1021/nl0808076. 18700806

[ref25] Garnett E.; Yang P. Light Trapping in Silicon Nanowire Solar Cells. Nano Lett. 2010, 10, 1082–1087. 10.1021/nl100161z. 20108969

[ref26] Wen L.; Zhao Z.; Li X.; Shen Y.; Guo H.; Wang Y. Theoretical Analysis and Modeling of Light Trapping in High Efficicency GaAs Nanowire Array Solar Cells. Appl. Phys. Lett. 2011, 99, 143116 10.1063/1.3647847.

[ref27] Zhou B.; Kong X.; Vanka S.; Chu S.; Ghamari P.; Wang Y.; Pant N.; Shih I.; Guo H.; Mi Z. Gallium Nitride Nanowire as a Linker of Molybdenum Sulfides and Silicon for Photoelectrocatalytic Water Splitting. Nat. Commun. 2018, 9, 3856 10.1038/s41467-018-06140-1. 30242212 PMC6155116

[ref28] Standing A.; Assali S.; Gao L.; Verheijen M. A.; Van Dam D.; Cui Y.; Notten P. H. L.; Haverkort J. E. M.; Bakkers E. P. A. M. Efficient Water Reduction with Gallium Phosphide Nanowires. Nat. Commun. 2015, 6, 7824 10.1038/ncomms8824. 26183949 PMC4518318

[ref29] Alqahtani M.; Sathasivam S.; Alhassan A.; Cui F.; Benjaber S.; Blackman C.; Zhang B.; Qin Y.; Parkin I. P.; Nakamura S.; Liu H.; Wu J. InGaN/GaN Multiple Quantum Well Photoanode Modified with Cobalt Oxide for Water Oxidation. ACS Appl. Energy Mater. 2018, 1, 6417–6424. 10.1021/acsaem.8b01387.

[ref30] Alqahtani M.; Ben-Jabar S.; Ebaid M.; Sathasivam S.; Jurczak P.; Xia X.; Alromaeh A.; Blackman C.; Qin Y.; Zhang B.; Ooi B. S.; Liu H.; Parkin I. P.; Wu J. Gallium Phosphide Photoanode Coated with TiO 2 and CoO x for Stable Photoelectrochemical Water Oxidation. Opt. Express 2019, 27, A364 10.1364/oe.27.00a364. 31052888

[ref31] Wu J.; Li Y.; Kubota J.; Domen K.; Aagesen M.; Ward T.; Sanchez A.; Beanland R.; Zhang Y.; Tang M.; Hatch S.; Seeds A.; Liu H. Wafer-Scale Fabrication of Self-Catalyzed 1.7 EV GaAsP Core-Shell Nanowire Photocathode on Silicon Substrates. Nano Lett. 2014, 14, 2013–2018. 10.1021/nl500170m. 24679049

[ref32] Moehl T.; Suh J.; Sévery L.; Wick-Joliat R.; Tilley S. D. Investigation of (Leaky) ALD TiO2 Protection Layers for Water-Splitting Photoelectrodes. ACS Appl. Mater. Interfaces 2017, 9, 43614–43622. 10.1021/acsami.7b12564. 29190064

[ref33] Chu S.; Vanka S.; Wang Y.; Gim J.; Wang Y.; Ra Y. H.; Hovden R.; Guo H.; Shih I.; Mi Z. Solar Water Oxidation by an InGaN Nanowire Photoanode with a Bandgap of 1.7 EV. ACS Energy Lett. 2018, 3, 307–314. 10.1021/acsenergylett.7b01138.

[ref34] Varadhan P.; Fu H. C.; Kao Y. C.; Horng R. H.; He J. H. An Efficient and Stable Photoelectrochemical System with 9% Solar-to-Hydrogen Conversion Efficiency via InGaP/GaAs Double Junction. Nat. Commun. 2019, 10, 5282 10.1038/s41467-019-12977-x. 31754117 PMC6872648

[ref35] Mattelaer F.; Vereecken P. M.; Dendooven J.; Detavernier C. The Influence of Ultrathin Amorphous ALD Alumina and Titania on the Rate Capability of Anatase TiO2 and LiMn2O4 Lithium Ion Battery Electrodes. Adv. Mater. Interfaces 2017, 4, 1601237 10.1002/admi.201601237.

[ref36] Memarzadeh Lotfabad E.; Kalisvaart P.; Cui K.; Kohandehghan A.; Kupsta M.; Olsen B.; Mitlin D. ALD TiO2 Coated Silicon Nanowires for Lithium Ion Battery Anodes with Enhanced Cycling Stability and Coulombic Efficiency. Phys. Chem. Chem. Phys. 2013, 15, 13646–13657. 10.1039/c3cp52485j. 23836149

[ref37] Yang X.; Liu R.; Du C.; Dai P.; Zheng Z.; Wang D. Improving Hematite-Based Photoelectrochemical Water Splitting with Ultrathin TiO2 by Atomic Layer Deposition. ACS Appl. Mater. Interfaces 2014, 6, 12005–12011. 10.1021/am500948t. 25069041

[ref38] Imrich T.; Zazpe R.; Krýsová H.; Paušová; Dvorak F.; Rodriguez-Pereira J.; Michalicka J.; Man O.; Macak J. M.; Neumann-Spallart M.; Krýsa J. Protection of Hematite Photoelectrodes by ALD-TiO2 Capping. J. Photochem. Photobiol., A 2021, 409, 113126 10.1016/j.jphotochem.2020.113126.

[ref39] Zazpe R.; Prikryl J.; Gärtnerova V.; Nechvilova K.; Benes L.; Strizik L.; Jäger A.; Bosund M.; Sopha H.; Macak J. M. Atomic Layer Deposition Al2O3 Coatings Significantly Improve Thermal, Chemical, and Mechanical Stability of Anodic TiO2 Nanotube Layers. Langmuir 2017, 33, 3208–3216. 10.1021/acs.langmuir.7b00187. 28291942 PMC5382572

[ref40] Ng S.; Sopha H.; Zazpe R.; Spotz Z.; Bijalwan V.; Dvorak F.; Hromadko L.; Prikryl J.; Macak J. M. TiO2 ALD Coating of Amorphous TiO2 Nanotube Layers: Inhibition of the Structural and Morphological Changes Due to Water Annealing. Front. Chem. 2019, 7, 38 10.3389/fchem.2019.00038. 30775363 PMC6367259

[ref41] Choi S.; Hwang J.; Lee T. H.; Kim H. H.; Hong S. P.; Kim C.; Choi M. J.; Park H. K.; Bhat S. S. M.; Suh J. M.; Lee J.; Choi K. S.; Hong S. H.; Shin J. C.; Jang H. W. Photoelectrochemical Hydrogen Production at Neutral PH Phosphate Buffer Solution Using TiO2 Passivated InAs Nanowire/p-Si Heterostructure Photocathode. Chem. Eng. J. 2020, 392, 123688 10.1016/j.cej.2019.123688.

[ref42] Li L. F.; Li Y. F.; Liu Z. P. CO2 Photoreduction via Quantum Tunneling: Thin TiO2-Coated GaP with Coherent Interface to Achieve Electron Tunneling. ACS Catal. 2019, 9, 5668–5678. 10.1021/acscatal.9b01645.

[ref43] Hu S.; Shaner M. R.; Beardslee J. A.; Lichterman M.; Brunschwig B. S.; Lewis N. S. Amorphous TiO2 Coatings Stabilize Si, GaAs, and GaP Photoanodes for Efficient Water Oxidation. Science 2014, 344, 1005–1009. 10.1126/science.1251428. 24876492

[ref44] Scheuermann A. G.; Prange J. D.; Gunji M.; Chidsey C. E. D.; McIntyre P. C. Effects of Catalyst Material and Atomic Layer Deposited TiO2 Oxide Thickness on the Water Oxidation Performance of Metal-Insulator-Silicon Anodes. Energy Environ. Sci. 2013, 6, 2487–2496. 10.1039/c3ee41178h.

[ref45] Cao S.; Kang Z.; Yu Y.; Du J.; German L.; Li J.; Yan X.; Wang X.; Zhang Y. Tailored TiO2 Protection Layer Enabled Efficient and Stable Microdome Structured P-GaAs Photoelectrochemical Cathodes. Adv. Energy Mater. 2020, 10, 1902985 10.1002/aenm.201902985.

[ref46] Gu J.; Yan Y.; Young J. L.; Steirer K. X.; Neale N. R.; Turner J. A. Water Reduction by a P-GaInP2 Photoelectrode Stabilized by an Amorphous TiO2 Coating and a Molecular Cobalt Catalyst. Nat. Mater. 2016, 15, 456–460. 10.1038/nmat4511. 26689139

[ref47] Su J.; Wei Y.; Vayssieres L. Stability and Performance of Sulfide-, Nitride-, and Phosphide-Based Electrodes for Photocatalytic Solar Water Splitting. J. Phys. Chem. Lett. 2017, 8, 5228–5238. 10.1021/acs.jpclett.7b00772. 28972772

[ref48] Zhang Y.; Aagesen M.; Holm J. V.; Jørgensen H. I.; Wu J.; Liu H. Self-Catalyzed GaAsP Nanowires Grown on Silicon Substrates by Solid-Source Molecular Beam Epitaxy. Nano Lett. 2013, 13, 3897–3902. 10.1021/nl401981u. 23899047

[ref49] Zhang Y.; Fonseka H. A.; Aagesen M.; Gott J. A.; Sanchez A. M.; Wu J.; Kim D.; Jurczak P.; Huo S.; Liu H. Growth of Pure Zinc-Blende GaAs(P) Core-Shell Nanowires with Highly Regular Morphology. Nano Lett. 2017, 17, 4946–4950. 10.1021/acs.nanolett.7b02063. 28758401

[ref50] Anitha V. C.; Zazpe R.; Krbal M.; Yoo J. E.; Sopha H.; Prikryl J.; Cha G.; Slang S.; Schmuki P.; Macak J. M. Anodic TiO2 Nanotubes Decorated by Pt Nanoparticles Using ALD: An Efficient Electrocatalyst for Methanol Oxidation. J. Catal. 2018, 365, 86–93. 10.1016/j.jcat.2018.06.017.

[ref51] Alotaibi B.; Nguyen H. P. T.; Zhao S.; Kibria M. G.; Fan S.; Mi Z. Highly Stable Photoelectrochemical Water Splitting and Hydrogen Generation Using a Double-Band InGaN/GaN Core/Shell Nanowire Photoanode. Nano Lett. 2013, 13, 4356–4361. 10.1021/nl402156e. 23927558

[ref52] Miao W.; Yao Y.; Zhang Z.; Ma C.; Li S.; Tang J.; Liu H.; Liu Z.; Wang D.; Camburn M. A.; Fang J. C.; Hao R.; Fang X.; Zheng S.; Hu N.; Wang X. Micro-/Nano-Voids Guided Two-Stage Film Cracking on Bioinspired Assemblies for High-Performance Electronics. Nat. Commun. 2019, 10, 3862 10.1038/s41467-019-11803-8. 31455776 PMC6711965

[ref53] Zhang Y.; Sanchez A. M.; Sun Y.; Wu J.; Aagesen M.; Huo S.; Kim D.; Jurczak P.; Xu X.; Liu H. Influence of Droplet Size on the Growth of Self-Catalyzed Ternary GaAsP Nanowires. Nano Lett. 2016, 16, 1237–1243. 10.1021/acs.nanolett.5b04554. 26708002

[ref54] Wilson R. L.; Simion C. E.; Blackman C. S.; Carmalt C. J.; Stanoiu A.; Di Maggio F.; Covington J. A. The Effect of Film Thickness on the Gas Sensing Properties of Ultra-Thin TiO2 Films Deposited by Atomic Layer Deposition. Sensors 2018, 18, 735 10.3390/s18030735. PMC5876708 29494504

[ref55] Li Y.; Zhang L.; Torres-Pardo A.; González-Calbet J. M.; Ma Y.; Oleynikov P.; Terasaki O.; Asahina S.; Shima M.; Cha D.; Zhao L.; Takanabe K.; Kubota J.; Domen K. Cobalt Phosphate-Modified Barium-Doped Tantalum Nitride Nanorod Photoanode with 1.5% Solar Energy Conversion Efficiency. Nat. Commun. 2013, 4, 2566 10.1038/ncomms3566. 24089138

[ref56] Chen S.; Takata T.; Domen K. Particulate Photocatalysts for Overall Water Splitting. Nat. Rev. Mater. 2017, 2, 17050 10.1038/natrevmats.2017.50.

[ref57] Promdet P.; Quesada-Cabrera R.; Sathasivam S.; Li J.; Jiamprasertboon A.; Guo J.; Taylor A.; Carmalt C. J.; Parkin I. P. High Defect Nanoscale ZnO Films with Polar Facets for Enhanced Photocatalytic Performance. ACS Appl. Nano Mater. 2019, 2, 2881–2889. 10.1021/acsanm.9b00326.

[ref58] Ling M.; Blackman C. Growth Mechanism of Planar or Nanorod Structured Tungsten Oxide Thin Films Deposited via Aerosol Assisted Chemical Vapour Deposition (AACVD). Phys. Status Solidi C 2015, 12, 869–877. 10.1002/pssc.201510047.

[ref59] Xia X.; Taylor A.; Zhao Y.; Guldin S.; Blackman C. Use of a New Non-Pyrophoric Liquid Aluminum Precursor for Atomic Layer Deposition. Materials 2019, 12, 1429 10.3390/ma12091429. PMC6540254 31052512

[ref60] Ferrero G. A.; Preuss K.; Marinovic A.; Jorge A. B.; Mansor N.; Brett D. J. L.; Fuertes A. B.; Sevilla M.; Titirici M. M. Fe-N-Doped Carbon Capsules with Outstanding Electrochemical Performance and Stability for the Oxygen Reduction Reaction in Both Acid and Alkaline Conditions. ACS Nano 2016, 10, 5922–5932. 10.1021/acsnano.6b01247. 27214056

[ref61] Channa A. I.; Tong X.; Xu J. Y.; Liu Y.; Wang C.; Sial M. N.; Yu P.; Ji H.; Niu X.; Wang Z. M. Tailored Near-Infrared-Emitting Colloidal Heterostructured Quantum Dots with Enhanced Visible Light Absorption for High Performance Photoelectrochemical Cells. J. Mater. Chem. A 2019, 7, 10225–10230. 10.1039/c9ta01052a.

