# Supplementary material for: Robust Protection of III–V Nanowires in Water Splitting by a Thin Compact TiO2 Layer
Source: ACS Appl Mater Interfaces. 2021 Jun 23;13(26):30950–8. doi: 10.1021/acsami.1c03903 (PMC8289235; doi:10.1021/acsami.1c03903)
Supplement: Supplementary file 2 — am1c03903_si_002.pdf [file am1c03903_si_002.pdf]

Supporting Information

Robust Protection of III-V Nanowires in Water Splitting by a Thin Compact TiO_2 Layer

Fan Cui[1†], Yunyan Zhang[1,8†*], H. Aruni Fonseka[2], Premrudee Promdet[3], Ali Imran Channa[4], Mingqing Wang[5], Xueming Xia[3], Sanjayan Sathasivam[3], Hezhuang Liu[4], Ivan P. Parkin[3], Hui Yang[6], Ting Li[7], Kwang-Leong Choy[5*], Jiang Wu[4*], Christopher Blackman[3], Ana M. Sanchez[2] & Huiyun Liu[1]

1. Department of Electronic and Electrical Engineering, University College London, London WC1E 7JE, UK
2. Department of Physics, University of Warwick, Coventry CV4 7AL, United Kingdom
3. Department of Chemistry, University College London, London WC1H 0AJ, UK
4. Institute of Fundamental and Frontier Sciences, University of Electronic Science and Technology of China, Chengdu 610054, P. R. China
5. UCL Institute for Materials Discovery, University College London, Roberts Building, Malet Place, London, WC1E 7JE, UK.
6. Department of Materials, Imperial College London, Exhibition Road, London SW7 2AZ, UK
7. Institute of Biomedical Engineering, Chinese Academy of Medical Sciences & Pecking Union Medical College, Tianjin, 300192
8. Department of Physics, Universität Paderborn, Warburger Straße 100, 33098, Paderborn, Germany

† These authors contributed equally to this work.

E-mail: yunyan.zhang.11@ucl.ac.uk, k.choy@ucl.ac.uk, jiangwu@uestc.edu.cn

1. Chemical properties of TiO_2

X-ray photoelectron spectroscopy (XPS) spectra of Titanium oxide protection layer before the photoelectrochemical (PEC) water splitting reaction is shown in Figure S1a. The deposited thin film exhibits binding energy peaks of Ti $2p_{1/2}$ and Ti $2p_{3/2}$ at 464.0 eV and 458.4 eV, respectively, corresponding to Ti^{4+} states in TiO_2.

There is no peak position shift observed from both Ti 2p spectra before and after the PEC reaction, which suggests that there are no noticeable changes in chemical format of TiO_2 films during PEC water splitting.

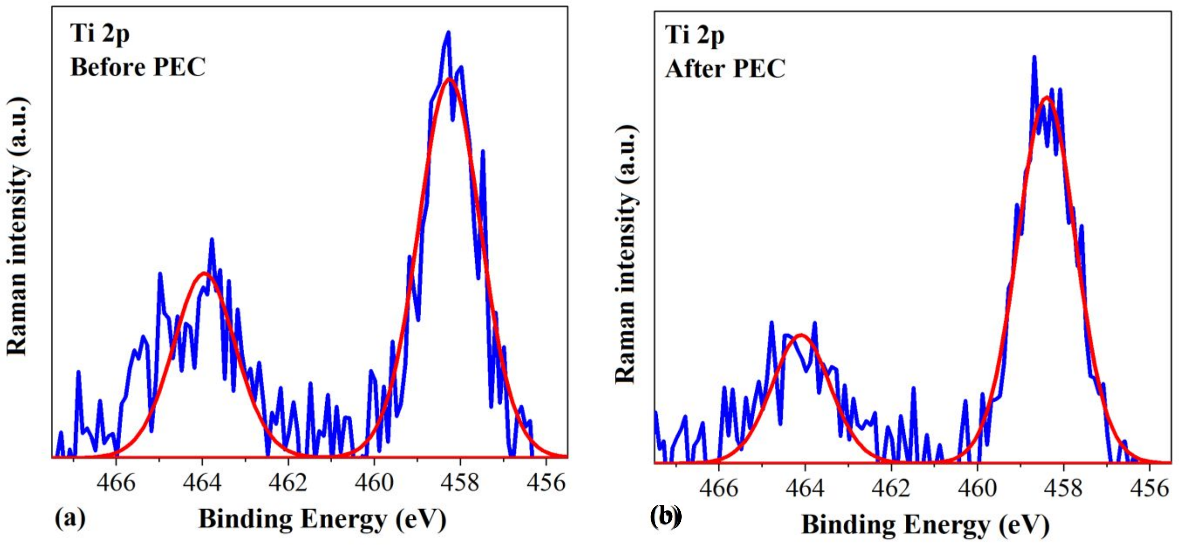

Figure S1 High-resolution XPS spectra of Ti 2p **(a)** before and **(b)** after 67-hour PEC test.

2. Amorphous structure of TiO_2

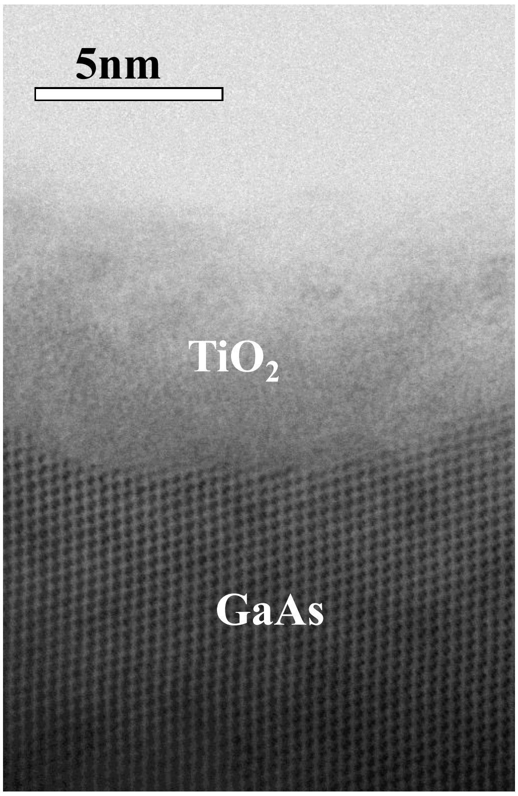

Figure S2 High-resolution TEM image clearly shows the crystal lattice of GaAs and the amorphous TiO_2.

3. H_2 generation during PEC reaction

There are apparent hydrogen generation during the stability test, which is carried out under 0.6 V vs RHE. It can be observed that hydrogen is uniformly generated on the surface of the test sample and the hydrogen generation rate is fast and stable.

(Double click to check the hydrogen generation video.)

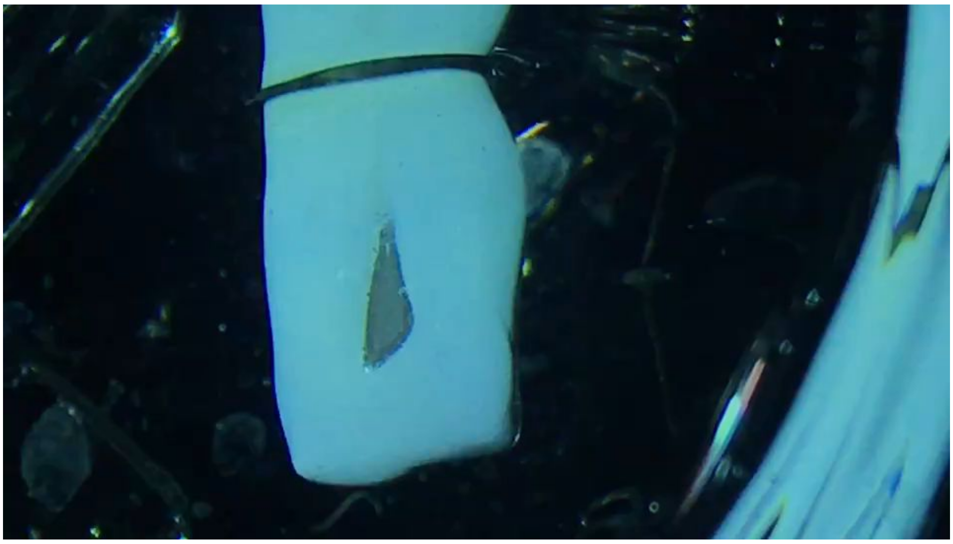

Video S1. hydrogen generation during the stability test,

4. Device state before and after PEC reaction

After 67-hour PEC reaction, the epoxy was ripped open at the rim of the sample, which caused the leak in of electrolyte solution to the back contract, and hence the failure of the device.

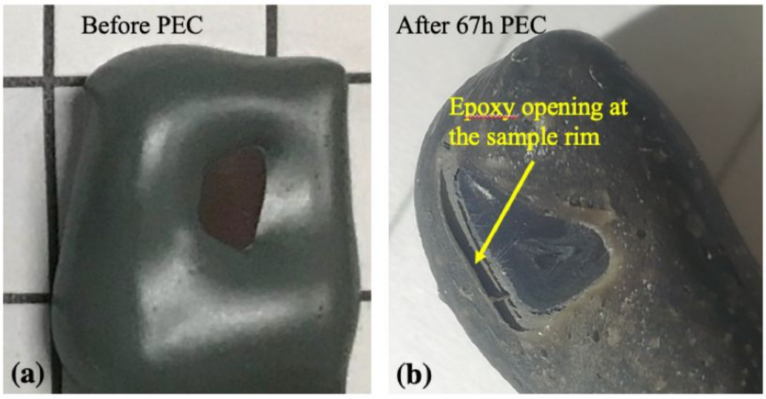

Figure S3 The topography of the photoelectrodes **(a)** before and **(b)** after stability test. The epoxy failed under 67 hours of corrosion, resulting in contact failure.
